# Ten years of proficiency testing reveals an improvement in the analytical performance of EU National Reference Laboratories for genetically modified food and feed

**DOI:** 10.1016/j.foodcont.2020.107237

**Published:** 2020-08

**Authors:** Wim Broothaerts, Fernando Cordeiro, Piotr Robouch, Hendrik Emons

**Affiliations:** European Commission, Joint Research Centre (JRC), Geel, Belgium

**Keywords:** Proficiency test results, Genetically modified food, National reference laboratory, Long-term performance

## Abstract

National Reference Laboratories (NRLs) in the Member States of the European Union (EU) monitor the implementation of the EU legislation on the presence of genetically modified organisms (GMOs) in food and feed. The EU Reference Laboratory for GM Food and Feed (EURL GMFF) supports the harmonisation of measurement procedures and the improvement of the analytical performance of these laboratories, among others through the organisation of a proficiency testing (PT) scheme. The PT results reported over 10 years have been analysed using common criteria applied to the reported data. The outcome revealed a gradual decrease of the relative standard deviation within the sets of the reported data with time. The extent of the deviation of the results from the assigned value also diminished between 2010 and 2019. The average deviation from the assigned value was independent of the GM content in the later PT rounds but it was affected by the complexity of the test item matrix. Performance scores were calculated for all results reported by the 62 NRLs. The number of unsatisfactory performance scores obtained decreased with time. The trends observed indicate an improvement in the analytical performance and an increased harmonisation of GMO testing within the EU enforcement laboratories.

## Highlights

• The reported results during 10 years of a proficiency testing scheme were evaluated.
• Common performance parameters were applied to visualise performance trends.
• The variability of the results around the assigned value decreased with time.
• The performance of 62 National Reference Laboratories improved with time.
• An increased harmonisation and accuracy of GMO testing was observed.

## 1 Introduction

The European Union introduced in 2003 a legal framework for the regulation of the presence of genetically modified organisms (GMOs) in food and feed. GMOs are required to be authorised following a risk assessment and an EU decision and their presence in food or feed products on the market needs to be monitored (EC, 2003). This monitoring aims to detect the occurrence of non-authorised GMOs and to identify and quantify the content of authorised GMOs in the products on the market. Products containing authorised GMOs in an amount above 0.9% mass fraction (m/m %) per individual ingredient need to be labelled as “*containing or produced from GMOs”*. This labelling intends to inform the consumer and provides them a purchasing choice. Monitoring is also performed for the detection of GMOs subject to pending (or expired) authorisations, for which the adventitious or technically unavoidable presence in feed is allowed up to a level of 0.1 m/m % (EU, 2011).

Regulation (EC) No 882/2004 (EC, 2004), now replaced by Regulation (EU) 2017/625 (EU, 2017), had introduced an EU-wide control system to protect the safety of our food and its compliance to various food and feed laws, including for GMOs. Every EU Member State (MS) is required to designate one or more enforcement ‘National Reference Laboratories' (NRLs under Regulation (EU) 2017/625, or NRL/625 in short) and, if needed, official (control) laboratories (OCLs) to perform the analytical tests on samples collected by their respective National Competent Authority. An EU Reference Laboratory for GM Food and Feed (EURL GMFF) was set up to “*contribute to the improvement and harmonisation of methods of analysis, test or diagnosis to be used by official laboratories/ … /and of the analytical, testing and diagnostic data generated by them*” (EU, 2017). The EURL was tasked among others (i) to provide details of analytical methods to be used by NRLs and (ii) to coordinate their application by organising proficiency testing (PT) rounds, (iii) to conduct training courses for the benefit of NRLs, and (iv) to provide assistance to the European Commission in case of contested analysis results or notifications of unauthorised GMOs that entered the EU market.

The EURL GMFF was legally assigned to the Joint Research Centre of the European Commission (EC, 2004; EU, 2017). The EURL GMFF has also the legal mandate (EC, 2003) for validating the detection methods for GMOs as part of their authorisation process. The interlaboratory validations are performed with support of NRLs who are selected from a list in an Annex to Regulation (EU) No 120/2014 (EU, 2014). While almost all enforcement NRLs (NRL/625) also act as NRL under the latter Regulation, there are 25 other laboratories who function only as NRL/120 (they are often also appointed as OCL by their Member State). In the present work, both NRL categories (NRL/625 and NRL/120) are usually considered together, unless otherwise indicated.

In parallel with the introduction of the GMO legislation, the capacity building in the EU Member States for taking care of the control tasks was driven through intense networking and over 20 EU-wide trainings have been organised by the EURL GMFF since 2000. A European Network of GMO Laboratories (ENGL) was set up in 2002 for supporting the EURL GMFF in the development and dissemination of best practices in the area of GMO testing (Emons, 2019).

In 2010 the EURL GMFF started to organise a PT scheme for the monitoring and improvement of the analytical competence of the official GMO control laboratories in the EU. Between 45 and 62 NRLs have participated to this scheme over the years. This number varied somewhat over time due to the nomination or denomination of NRLs by the Competent Authorities of the Member States. Two PT rounds have been organised per year since 2010, each including two test items to be analysed for the identity and quantity of one or more GM events. The characteristics of these test matrices changed with time. In 2010–2011 the test items were based on a uniform ground seed matrix, whereas from 2014 onwards, one test item in each PT round consisted of an easier ground seed matrix, while the other test item had a more challenging matrix (e.g. processed food, compound feed). The preparation, handling and distribution of the PT materials were performed in line with ISO/IEC 17043:2010 (ISO, 2010). Laboratory performance was evaluated by calculating ‘*z* scores' and reported and discussed in PT reports. NRLs that obtained an unsatisfactory *z* score were requested to perform a root-cause analysis and, when required, further support and training was provided by the EURL GMFF to avoid systematic errors.

This paper presents the evaluation of the long-term performance of the NRLs participating to the PT scheme of the EURL GMFF based on the analysis of reported results (expressed in GM mass fraction or DNA copy number ratio) for a total of 62 measurands in 40 test items. As the 86 datasets analysed generally appeared to be normally distributed (Broothaerts, Cordeiro, Corbisier, Robouch, & Emons, 2020), the reported data were evaluated on the raw scale *per se*, with no further logarithmic transformation as done in the respective PT rounds up to 2018. This approach allows comparison of the variation in the results and visualisation of the evolution of the laboratory performances over the years.

## 2 Materials and methods

### 2.1 Proficiency testing approaches

The EURL GMFF is ISO/IEC 17043:2010 accredited (ISO, 2010), fulfilling the general requirements for the competence of providers of proficiency testing schemes and for the development and operation of such exercises. It has organised a total of 20 PT rounds between 2010 and 2019. For each PT round two test items were prepared and distributed to the participants, consisting of a food or feed matrix containing or spiked with one or more GMOs. Invited participants registered online and received the test items together with the instruction to identify the unknown GM event(s) present in the materials and to quantify the event(s) identified. Each PT round involved between 63 and 102 official laboratories, including 45 to 62 EU NRLs (although not all of them reported results for each GM event). The analytical results and answers to a questionnaire were submitted online. The results were reported either in GM mass fraction (m/m %) or in DNA copy number ratio (cp/cp %, only until 2013). When the same laboratory had provided two results for the same analytical task, i.e. expressed in different units, only the result in m/m % was retained. Broothaerts et al. (2020) had shown that the EURL GMFF PT datasets were generally normally distributed, obviating the need for a log_10_-data transformation. Hence the results reported on the linear scale were used for the calculations performed in the present work. The relative standard deviation (RSD%) of each dataset was derived from the standard deviation divided by the mean of the results within the respective dataset.

The first 5 PT rounds included relatively simple test item matrices composed of 100% ground maize or soybean flour (PT1-4) or already extracted genomic DNA of maize and oilseed rape (PT5). In PT6, a compound feedstuff was mixed with ground maize and soybean (in a mass ratio of approximately 1:1:1) and spiked with a GM maize and a GM soybean event. In the following PT (PT7), biscuits were baked from 50% (m/m) wheat and 50% (m/m) maize flour (and eggs, butter and sugar) and spiked with three GM maize events. In PT8, rice noodles were mixed with 20% (m/m) soybean containing a soybean GM event.

In all the PT rounds until the end of 2013, the two test items prepared for each PT round were composed of the same sample matrix and the same GM events, but the GM mass fractions in both test items varied around the GM labelling threshold of 0.9 m/m %. In subsequent PT rounds (2014–2019), two different test items were produced for every PT round, the first one composed of a compound or processed food or feed (T1), the second one (T2) as a simple matrix being either 100% maize or 100% soybean (or a mixture of maize and soybean in PT18).

### 2.2 Assigned value

The assigned value (*x*_*pt*_) for each measurand (i.e. the content of a specific GM event in a given test item) was calculated in line with ISO 13528:2015 (ISO, 2015). It was determined in different ways in the past PT rounds, i.e. (i) from the formulation (mass fraction), (ii) by consensus of results reported by expert laboratories (using real-time or digital PCR), (iii) using the certified value of a certified reference material (CRM), or (iv) by calculating the robust mean of the results reported by the NRLs. The approach for calculating *x*_*pt*_ used in this study was identical to what has been reported in the respective PT reports (see http://gmo-crl.jrc.ec.europa.eu/Proficiency-tests.html), except that in this study robust statistics was applied to the results of NRLs only instead of to all reported results.

For (homozygous) soybean GM events, the results reported in m/m % and cp/cp % are comparable and were combined in a single dataset per measurand. When the number of results reported was below 5, which occurred for two datasets in cp/cp % for a (hemizygous) maize GM event, the assigned value was approximated as the robust mean derived from the results reported in m/m % for the same measurand divided by two (EURL GMFF, 2011).

### 2.3 Performance evaluation

The results reported by the NRLs were then compared to the assigned values and scored by using the percentage deviation (*D*_*i*_*%* = 100 (*x*_*i*_ – *x*_*pt*_)/*x*_*pt*_), in line with ISO 13528:2015, Section 9.3.1 (ISO, 2015). This parameter is easily understandable, does not require a predefined standard deviation for PT (*σ*_*pt*_), as used for calculating *z* scores, and is suitable for comparing the laboratory performance across PT rounds of different difficulty.

## 3 Results and discussion

### 3.1 Evolution of data variability

The reported results analysed in this study comprise 2921 unique quantitative measurement data, expressed in m/m % or cp/cp %, for 62 measurands in 40 test items almost equally divided over 20 PT rounds organised between 2010 and 2019 (Table 1). This resulted in 86 independent datasets of which 62 (72%) consisted of results expressed in m/m % or in both units (for GM soybean only) and 24 (28%) with results expressed in cp/cp %.

**Table 1:** Characteristics of the proficiency testing rounds organised by the EURL GMFF.

Year	PT round	Test Item	Matrix	GM event	Unit^a^	*x*_*pt*_^b^	*N*	RSD%
2010	PT1	1	100% Maize	1. NK603	m	0,10 f	29	60%
					cp	0,09 c	17	159%
		2	100% Maize	2. NK603	m	1,69 f	40	33%
					cp	1,49 c	19	132%
	PT2	3	100% Maize	3. MON810	m	0,81 f	41	58%
					cp	0,63 c	14	31%
		4	100% Maize	4. MON810	m	3,83 f	41	35%
					cp	2,70 c	13	35%
2011	PT3	5	100% Soybean	5. 40-3-2	m & cp	1,12 c	54	22%
		6	100% Soybean	6. 40-3-2	m & cp	3,33 c	52	20%
	PT4	7	100% Maize	7a. GA21	m	0,26 x	47	54%
					cp	0,14 x	12	105%
				7b. 1507	m	0,30 x	44	74%
					cp	0,19 x	15	88%
				7c. MIR604	m	3,38 x	43	65%
					cp	1,34 x	14	63%
		8	100% Maize	8a. GA21	m/	2,08 x	46	65%
					cp	0,86 x	13	103%
				8b. 1507	m	0,89 x	43	83%
					cp	0,43 x	13	80%
				8c. MIR604	m	0,89 x	43	69%
					cp	0,34 x	10	66%
2012	PT5	9	DNA Maize & Oilseed rape	9a. 59122	m	0,87 x	37	56%
					cp	0,48 x	19	60%
				9b. GT73	m	0,90 x	34	24%
					cp	0,39 x	20	117%
		10	DNA Maize & Oilseed rape	10a. 59122	m	3,61 x	36	37%
					cp	2,14 x	19	61%
				10b. GT73	m	0,39 x	31	90%
					cp	0,15 x	21	131%
	PT6	11	Animal feed/maize/soybean mix 1:1:1	11a. MON88017	m	0,68 x	39	45%
					cp	0,39 c	10	127%
				11b. 40-3-2	m & cp	1,78 x	50	125%
		12	Animal feed/maize/soybean mix 1:1:1	12a. MON88017	m	1,42 x	39	30%
					cp	0,72 c	10	98%
				12b. 40-3-2	m & cp	0,21 x	51	230%
2013	PT7	13	Baked biscuits	13a. 98140	m	0,33 c	40	58%
					cp	0,18 c	5	31%
				13b. MON810	m	0,92 c	40	44%
					cp	0,34 c	8	11%
				13c. MON863	m	1,66 c	43	69%
					cp	0,78 c	5	17%
		14	Baked biscuits	14a. 98140	m	1,06 c	40	60%
					cp	0,67 c	5	32%
				14b. MON810	m	0,36 c	41	54%
					cp	0,13 c	8	16%
				14c. MON863	m	0,69 c	43	71%
					cp	0,28 c	5	19%
	PT8	15	Rice noodles with 20% (m/m) soybean	15. 356043	m & cp	0,58 c	40	53%
		16	Rice noodles with 20% (m/m) soybean	16.356043	m & cp	1,34 c	41	33%
2014	PT9	17	Animal feed/maize/soybean mix 1:1:1	17a. NK603	m	0,84 c	40	31%
					cp	0,42 c/2	3	24%
				17b. MON88017	m	0,85 c	42	51%
					cp	0,42 c/2	3	27%
				17c. MON89788	m & cp	0,20 c	44	55%
				17d. 40-3-2	m & cp	1,02 c	46	19%
		18	100% Soybean	18. MON89788	m & cp	0,89 c	45	46%
	PT10	19	Chicken feed with 44% (m/m) soybean	19a. 40-3-2	m	0,94 c	43	63%
				19b. 40278	m	1,64 c	39	40%
		20	100% Maize	20. 40278	m	0,64 c	44	20%
2015	PT11	21	Rice noodles with 20% (m/m) soybean	21. 356043	m	1,35 c	39	38%
		22	100% Soybean	22. 68416	m	0,45 c	41	27%
	PT12	23	Instant soup	23. MON88302	m	1,20 c	39	37%
		24	100% Soybean	24. 81419	m	0,99 v	41	17%
2016	PT13	25	Mexican tortilla chips	25a. 1507	m	0,74 c	49	64%
				25b. MIR162	m	2,64 c	45	39%
		26	100% Maize	26. 40278	m	0,63 c	50	21%
	PT14	27	Rapeseed cake	27a. 73496	m	0,50 c	37	56%
				27b. GT73	m	0,30 c	45	155%
		28	100% Soybean	28. MON89788	m	0,83 c	47	39%
2017	PT15	29	Soya milk powder	29. DAS-44406	m	0,54 c	51	68%
		30	100% Maize	30. VCO-1981	m	1,00 v	49	21%
	PT16	31	Chicken feed with 8% (m/m) soybean	31. 40-3-2	m	0,81 c	52	29%
		32	100% Soybean	32. 40-3-2	m	0,77 c	54	20%
2018	PT17	33	Maize bread	33a. MON810	m	0,47 x	34	70%
				33b. MON89034	m	0,92 x	39	35%
		34	100% Soybean	34. 68416	m	0,42 x	46	19%
	PT18	35	Pig feed	35. 40-3-2	m	3,47 x	44	27%
		36	Maize/soybean mix 1:1	36a. Bt11	m	0,80 x	41	31%
				36b. MON87701	m	0,92 x	43	30%
2019	PT19	37	Maize tortilla chips	37. NK603	m	1,76 x	47	39%
		38	100% Maize	38. 4114	m	1,00 v	50	19%
	PT20	39	Animal feed	39a. 40-3-2	m	1,01 x	42	36%
				39b. MON87708	m	6,64 x	39	50%
				39c. GHB119	m	0,90 x	19	28%
		40	100% Soybean	40. DAS-44406	m	0,70 x	46	19%

a Unit of expression: m/m % (m) or cp/cp % (cp).

b Assigned value (*x*_*pt*_) derived from formulation (f), consensus of NRL results (c or c/2, see Materials and Methods), expert laboratories (x), or CRM certificate (v).

The analytical workflow followed in the laboratories included (i) the extraction of DNA, and (ii) the use of the extracted DNA in quantitative PCR (qPCR) for the GM event and for a taxon-specific endogenous reference target. The ratio of both qPCR results then represents the relative GM content. In order to establish the performance trends of enforcement NRLs in the PT rounds between 2010 and 2019 the characteristics of the test items investigated need to be taken into account (Table 1). The use of a more difficult test item challenges mainly the competence for extracting DNA of appropriate quality and quantity from a compound or processed material, similarly as required for the routine analysis of market products by the enforcement laboratories. While the difficulty of the PT has been gradually increased for test item T1 over the years, in parallel with the increased technical capabilities of the laboratories, the matrix characteristics of T2 remained relatively constant.

**Fig. 1:**
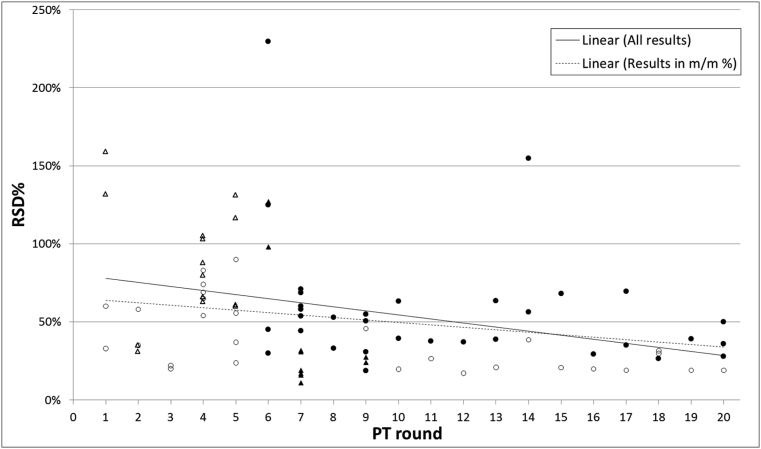
Variability of the reported results per dataset for each PT round, expressed as RSD%. Each symbol corresponds to one dataset expressed in m/m % (circles) or cp/cp % (triangles). Open and closed symbols refer to simple or challenging test item matrices, respectively.

The relative standard deviation (RSD%) has been calculated for each dataset as an expression of the variability of the results for a certain analyte in a test item (Table 1). Irrespective of the measurement unit of expression, RSD% seems to decrease over time (Fig. 1), which indicates that the spread of the results is smaller in the later PT rounds compared to the earlier ones. The largest variation is seen in the first 6 PT rounds, despite the fact that 5 of these included simple test matrices (open symbols in Fig. 1) composed of ground maize or soybean. In the later PT rounds, the RSD% is generally lower for the simple test matrices compared to the more challenging processed food or compound feed matrices (closed symbols in Fig. 1).

For the same measurand, the RSD% is often larger for results expressed in cp/cp % compared to those expressed in m/m %. Indeed, almost 1/3 of the results expressed in cp/cp % has an RSD% above 100%, compared to only 5% of the results in m/m %. This may be attributed to (i) the larger size of the datasets in m/m %, (ii) the smaller number of datasets in cp/cp %, which are only available for the earlier PT rounds, while those in m/m % are available for all PT rounds and these, therefore, may include a factor revealing the performance improvement over the years, and (iii) the use of inaccurate conversion factors (or ignorance of them) for the conversion of certified values of CRM calibrants expressed in m/m % into corresponding copy number ratios (Corbisier & Emons, 2019). For instance, the analyst did not properly take into account that the endogenous reference gene existed in more copies in the genome than the GM insert (e.g. *CruA* for oilseed rape; Jacchia et al., 2018) or that its quantification was biased (e.g. *Adh1*-70 basepairs; Broothaerts et al., 2008). In line with these explanations, all but two RSD% values above 70% (Fig. 1) are derived from measurements of GM maize or GM oilseed rape events, crops in which the mathematical relation between GM mass fractions and GM copy number ratios is not fixed because of biological peculiarities (Corbisier & Emons, 2019; Holst-Jensen, De Loose, & Van Den Eede, 2006).

### 3.2 Effect of test item matrix and PT year on the deviation from the assigned value

The assigned value (*x*_*pt*_) for each individual dataset was obtained from expert laboratories (i.e. the EURL GMFF) for 34 data sets (40%), from formulation (4 datasets) or from the certificate of a CRM (3 datasets), in line with the values reported in the respective PT reports issued twice per year. In the absence of such pre-determined values in the PT reports, *x*_*pt*_ was re-calculated in this study as consensus value of the NRL results. This was done for 45 datasets (52%), which included mainly those from PT rounds organised in 2013–2017.

**Fig. 2:**
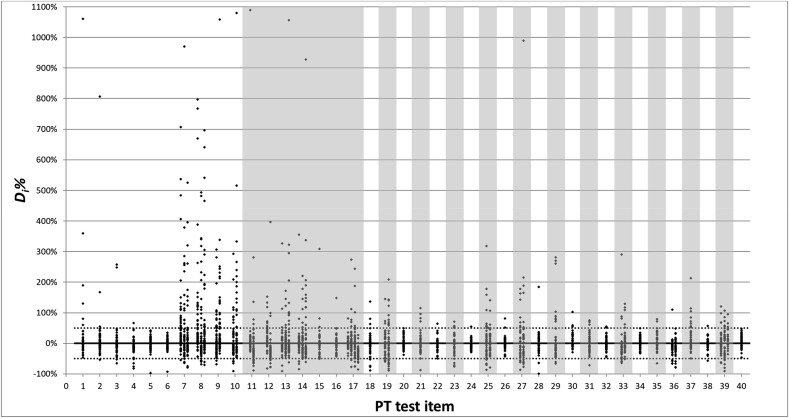
Deviation of the individual laboratory results from the assigned value per test item, expressed as *D*_*i*_*%*. Each column of results corresponds to a given measurand (note: one outlying value (2900%) was removed from the first column of test item 12). Dotted lines correspond to the 50% satisfaction limits. The more challenging test item matrices are gray shaded.

For each of the reported results the percentage difference (*D*_*i*_*%*; ISO, 2015) from the corresponding assigned value was calculated. This parameter is independent from the standard deviation for performance assessment (*σ*_*pt*_, which varied between PT rounds from 0.1 to 0.25 on the logarithmic scale), and allows comparing laboratory performances across PT rounds. This approach also enables to combine the results for each analyte expressed in either measurement unit of expression (m/m % or cp/cp %). Plotting the *D*_*i*_*%* for the sequential PT measurements confirms the trend towards a reduced scattering of the data over the ten years studied (Fig. 2). It can also be seen that the more challenging matrices used in a PT round (shaded areas in Fig. 2) generally stipulate larger deviating results compared to those reported for the easier (ground seed) test items.

### 3.3 Performance evaluation

**Fig. 3:**
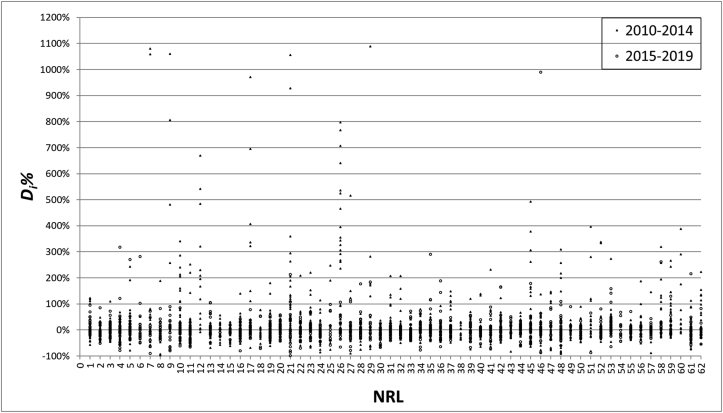
Laboratory performance per NRL expressed as deviation of the reported results from the assigned value for the period 2010–2014 (triangles) and 2015–2019 (circles).

The *D*_*i*_*%* values obtained by each NRL can also serve as competence indicator for the laboratory regarding the accuracy of GMO quantification in different sample matrices, and may reveal the evolution of this competence with time. The analytical performance of the 62 NRLs over the past ten years is shown in Fig. 3 for NRL/625 (N1 to N37) and NRL/120 (N38 to N62). Large differences in the dispersion of the reported results are observed between the NRLs. Most of the extreme results were derived from the earlier PT rounds (2010–2014) and often correspond to results expressed in copy number ratio.

Assuming a realistic *σ*_*pt*_ of 25% of the assigned value (Broothaerts et al., 2020), satisfactory, questionable and unsatisfactory results would be characterised by |*D*_*i*_*%*| ≤ 50%; 50% < |*D*_*i*_*%*| < 75%, and |*D*_*i*_*%*| ≥ 75%, respectively. The three performance categories, which are identical to the evaluation of *z* scores (ISO, 2010), were visually coded green, yellow and red, respectively, to evaluate the performance of each NRL based on the results reported over the period 2010–2019 (Table 2). The variation in the number of results reported (white cells in the table mean that no result was reported for that measurand) may be due to the fact that participation of NRL/120, in contrast to NRL/625, was not mandatory, or because some NRLs switched their NRL status at some moment in time. In addition, several test items were not analysed by a laboratory when the scope of its accreditation was exclusively for either food or feed, or when the corresponding detection method had not (yet) been verified in the laboratory. During the first four years a pattern of unsatisfactory scores is visible for results reported for the same measurand present at different mass fractions in the two test items of a single PT round (e.g. 7a-8a, 7b–8b, 9b–10b and 13c-14c in Table 2, numbers explained in Table 1). This may have been caused by problems of the laboratory to master the method appropriately or they made the same (calculation) mistake twice. The percentage of acceptable performance results (green and yellow cells) obtained over the ten years investigated varies between the NRLs from 70% to 100% (excluding three NRLs [N12, N38 and N60] who participated only during the early years of proficiency testing). Three NRLs got always satisfactory scores except for one (N55) or two (N20 and N33) questionable scores for 57 reported results or more (for a maximum number of 62 measurands). A few other NRLs reported only satisfactory or questionable results over the whole period, or obtained only one unsatisfactory score amongst a majority of satisfactory scores. Furthermore, the NRLs N37 and N50 reported systematically satisfactory results for all 26 measurands over the last 5 years, despite the increased complexity of some of the test items to be analysed. On the contrary, a few laboratories (N4, N35, N36) experienced a minor reverse trend in their performance, obtaining 3 or more unsatisfactory scores in the last 5 years but none before.

**Table 2:**
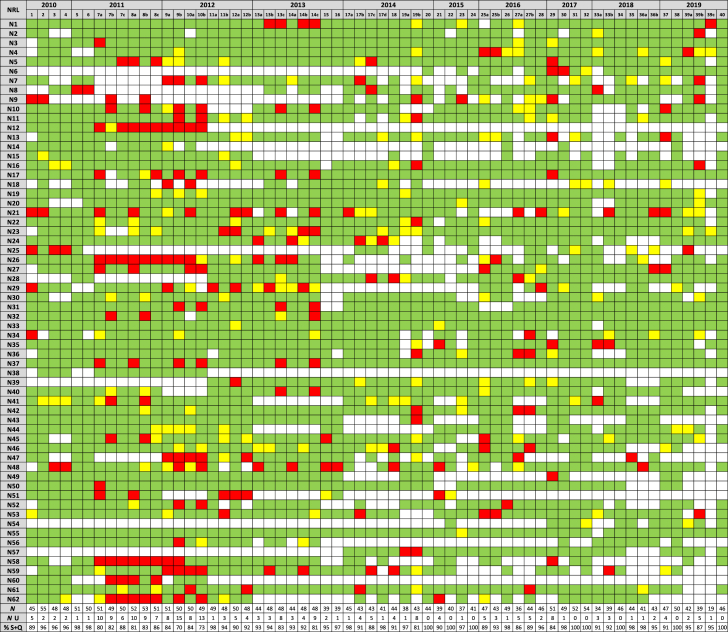
Performance evaluation per NRL between 2010 and 2019. Green = satisfactory (|*D*_*i*_*%*| ≤ 50%), yellow = questionable (50% < |*D*_*i*_*%*| < 75%), red = unsatisfactory (|*D*_*i*_*%*| ≥ 75%), white = no result reported. *N*, total number of results; *N* U, number of unsatisfactory results; % S + Q, percentage satisfactory and questionable results.

**Fig. 4:**
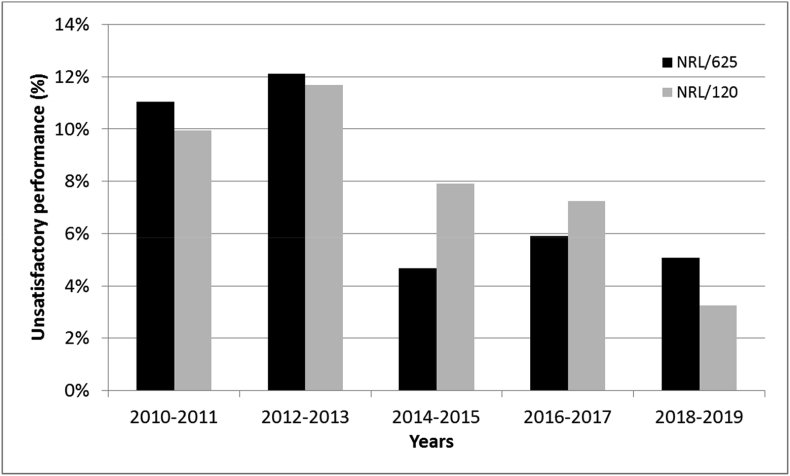
Evolution of the performance of NRL/625 and NRL/120 with time, expressed as percentage of unsatisfactory scores obtained per time window of two years.

The overall improvement in the performance of NRLs over the years is summarised in Fig. 4. The results are shown for both NRL/625 and NRL/120. While 10–12% of the reported results were considered unsatisfactory in the early years of running the PT scheme, this portion decreased significantly since 2014. The strongest improvement is seen for the NRL/120 who obtained only 3% of unsatisfactory performance scores in the last two years, slightly better than the NRL/625 (5%). Since 2014, the fraction of NRL/625 with 100% acceptable scores, i.e. without any unsatisfactory score, remained more or less stable around 60%, compared to over 80% for NRL/120. The difference between the two NRL categories is, however, not large because NRL/120 tend more easily to refrain from certain measurements and PT participations.

The acceptance rate of the results reported for the easier test items composed of ground seed or grain was higher (73% of NRLs had 100% satisfactory results in the period 2015–2019) than for the more challenging materials corresponding to processed or compound market products (19%). Whereas two third of the total number of reported results were obtained on the challenging test items, they accounted for 90% of all unsatisfactory scores attributed. This is mainly due to the difficulty in extracting representative DNA of good quality from the diverse food or feed products used as test item (Corbisier et al., 2007; Demeke & Jenkins, 2010). In addition, the challenging test items often contained more than one GM event (in crops such as maize, soybean or oilseed rape), in contrast to the easier test items that consistently contained only a single GM maize or GM soybean event (except for test item 36 which included one GM maize and one GM soybean event). Problems with DNA extraction from highly processed food or compound feed products are one of the most common issues identified during the root-cause analysis requested to the NRLs with an unsatisfactory performance. The extraction problem, more than the quantitative analysis itself, has been identified by several NRLs as requiring further guidance and training from the EURL GMFF in the coming years.

**Fig. 5:**
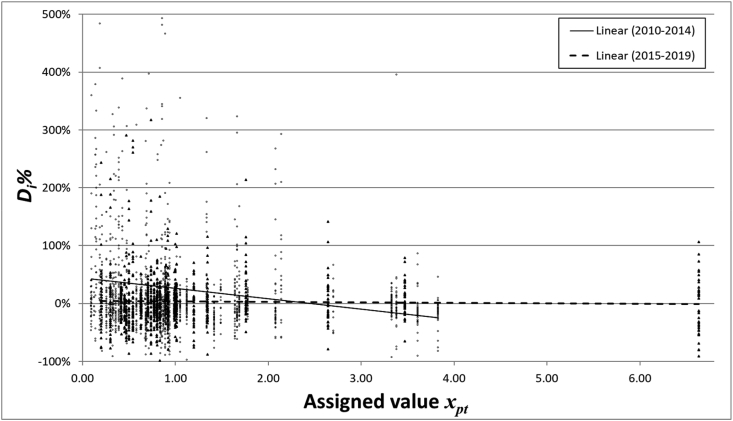
Variation of the reported results in function of the assigned value for the PT rounds of 2010–2014 (open circles, solid trendline) and 2015–2019 (closed triangles, dashed trendline). The Y axis scale is shown up to +500%.

In general, analytical measurements close to the limit of quantification (LOQ) are less accurate than measurements of analytes that are present in larger concentrations. In GMO testing by NRLs, measurements around the labelling threshold of 0.9 m/m % are most relevant, and these mass fractions were relatively more represented in the PT rounds. The GMO measurements revealed a decrease in *D*_*i*_*%* when the *x*_*pt*_ increases (Fig. 5). However, the trend towards an increased average accuracy of the measurement results for larger GMO content is only observed for the measurements in the early years of GM testing (2010–2014). In later years (2015–2019), the trend has disappeared, which indicates that GM testing has improved over the whole range of GMO mass fractions that are regularly assessed in the PT rounds.

## 4 Conclusions

The evolution of NRLs' performance in the PT scheme between 2010 and 2019 revealed the analytical improvement of the laboratories for GMO analysis over the years, and could be seen as an indicator of the usefulness and efficiency of the support provided by the EURL GMFF to the GMO control laboratories. NRL workshops have been organised annually, and guidance documents were issued by the EURL GMFF and the ENGL network (see e.g. http://gmo-crl.jrc.ec.europa.eu/guidancedocs.htm). An online GMO-Methods database was developed (Bonfini et al., 2012) and made publically available (http://gmo-crl.jrc.ec.europa.eu/gmomethods/). Similarly, training and advice were provided by the NRLs at national level to build up their national network of testing laboratories. Training, plenary meetings and workshops, guidance and networking gradually created a solid level playing field for the analytical competence of control laboratories and offered a platform for the exchange of experiences and solutions to analytical problems encountered (Emons, 2019). In addition, technical support was provided by the EURL GMFF to laboratories who had obtained an unsatisfactory performance score, in the frame of a follow-up process. This support highlighted methodological problems that were previously not well understood, resulting in their remediation and the application of improved procedures in the laboratories. The analytical performance of NRLs steadily increased over the years as a result of these targeted and multi-faceted actions.

## CRediT authorship contribution statement

**Wim Broothaerts:** Conceptualization, Investigation, Data curation, Writing - review & editing, Supervision. **Fernando Cordeiro:** Methodology, Software, Validation,Formal analysis, Writing - original draft. **Piotr Robouch:** Conceptualization, Validation, Writing - review & editing, Methodology. **Hendrik Emons:** Writing - review & editing.

## Declaration of competing interest

The authors declare that they have no conflict of interests.
